# Methyl pyrido[2,3-*b*]pyrazine-3-carboxyl­ate

**DOI:** 10.1107/S1600536811044412

**Published:** 2011-10-29

**Authors:** Hoong-Kun Fun, Madhukar Hemamalini, Anita Hazra, Shyamaprosad Goswami

**Affiliations:** a X-ray Crystallography Unit, School of Physics, Universiti Sains Malaysia, 11800 USM, Penang, Malaysia; b Department of Chemistry, Bengal Engineering and Science University, Shibpur, Howrah 711 103, India

## Abstract

The asymmetric unit of the title compound, C_9_H_7_N_3_O_2_, is composed of two independent mol­ecules. The crystal structure is stabilized by C—H⋯O and C—H⋯N hydrogen bonds, forming a three-dimensional network. The crystal structure also features pyrazine–pyrazine π–π inter­actions [centroid–centroid distance = 3.6994 (5) Å] and also pyridine–pyrazine π–π inter­actions [centroid–centroid distance = 3.6374 (5) Å].

## Related literature

For details of heterocyclic esters, see: Listvan *et al.* (2002 ▶); Li *et al.* (2007 ▶); Goswami & Hazra (2009 ▶); Goswami *et al.* (2011 ▶). For reference bond-length data, see: Allen *et al.* (1987 ▶). For the stability of the temperature controller used in the data collection, see: Cosier & Glazer (1986 ▶).
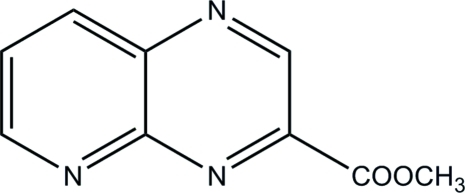


## Experimental

### 

#### Crystal data

C_9_H_7_N_3_O_2_
*M* *_r_* = 189.18
Monoclinic, 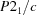

*a* = 9.5135 (1) Å
*b* = 26.9042 (3) Å
*c* = 6.7837 (1) Å
β = 107.686 (1)°
*V* = 1654.24 (4) Å^3^
*Z* = 8
Mo *K*α radiation
μ = 0.11 mm^−1^
*T* = 100 K
0.22 × 0.20 × 0.19 mm

#### Data collection

Bruker SMART APEXII CCD area-detector diffractometer
Absorption correction: multi-scan (*SADABS*; Bruker, 2009 ▶) *T*_min_ = 0.975, *T*_max_ = 0.979
18421 measured reflections
4876 independent reflections
4118 reflections with *I* > 2σ(*I*)
*R*_int_ = 0.025

#### Refinement

*R*[*F* ^2^ > 2σ(*F* ^2^)] = 0.041
*wR*(*F* ^2^) = 0.115
*S* = 1.04
4876 reflections
255 parameters
H-atom parameters constrained
Δρ_max_ = 0.38 e Å^−3^
Δρ_min_ = −0.30 e Å^−3^

### 

Data collection: *APEX2* (Bruker, 2009 ▶); cell refinement: *SAINT* (Bruker, 2009 ▶); data reduction: *SAINT*; program(s) used to solve structure: *SHELXTL* (Sheldrick, 2008 ▶); program(s) used to refine structure: *SHELXTL*; molecular graphics: *SHELXTL*; software used to prepare material for publication: *SHELXTL* and *PLATON* (Spek, 2009 ▶).

## Supplementary Material

Crystal structure: contains datablock(s) global, I. DOI: 10.1107/S1600536811044412/wn2453sup1.cif

Structure factors: contains datablock(s) I. DOI: 10.1107/S1600536811044412/wn2453Isup2.hkl

Supplementary material file. DOI: 10.1107/S1600536811044412/wn2453Isup3.cml

Additional supplementary materials:  crystallographic information; 3D view; checkCIF report

## Figures and Tables

**Table 1:** Hydrogen-bond geometry (Å, °)

*D*—H⋯*A*	*D*—H	H⋯*A*	*D*⋯*A*	*D*—H⋯*A*
C3*A*—H3*AA*⋯O2*A*^i^	0.93	2.54	3.2401 (13)	133
C4*A*—H4*AA*⋯N2*B*^ii^	0.93	2.55	3.3311 (14)	141
C9*A*—H9*AA*⋯N1*B*	0.96	2.62	3.4741 (14)	149
C3*B*—H3*BA*⋯O2*B*^iii^	0.93	2.53	3.2496 (14)	135
C4*B*—H4*BA*⋯N2*A*^iv^	0.93	2.51	3.3350 (14)	147
C9*B*—H9*BA*⋯N1*A*	0.96	2.57	3.4266 (14)	149

Symmetry codes: (i) 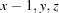; (ii) 
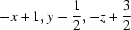
; (iii) 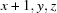; (iv) 
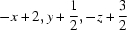
.

**Fig. 1.**
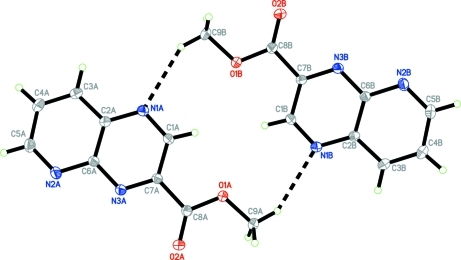
The asymmetric unit of the title compound, showing 50% probability displacement ellipsoids. Dashed lines indicate hydrogen bonds.

**Fig. 2.**
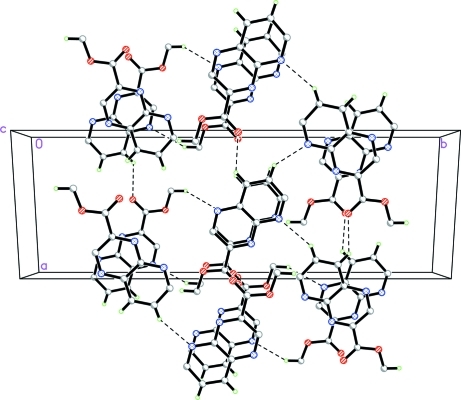
The crystal packing of the title compound. Dashed lines indicate hydrogen bonds.

## supplementary crystallographic information

### Comment

Heterocyclic esters are important compounds in respect of their biological and pharmaceutical characteristics (Listvan *et al.*, 2002; Li *et al.*, 2007). Recently we have developed a mild methodology for the synthesis of heterocyclic esters from their corresponding aldehydes (Goswami & Hazra, 2009; Goswami *et al.*, 2011). Here we report the crystal structure of methyl pyrido[2,3-b]pyrazine-3-carboxylate.

The asymmetric unit of the title compound consists of two crystallographically independent methyl pyrido[2,3-b]pyrazine-3-carboxylate molecules, (A & B), as shown in Fig. 1. The bond lengths of molecules A and B agree with each other and are within normal ranges (Allen *et al.*, 1987).

In the crystal structure (Fig. 2), the molecules are linked through intermolecular C—H···O and C—H···N hydrogen bonds (Table 1), forming a three-dimensional network. Furthermore, the crystal structure is stabilized by the following π–π interactions: (a) between pyrazine rings (N1A,N3A/C1A,C2A/C6A,C7A, centroid Cg1) Cg1···Cg1(1-x, -y, 1-z) 3.6994 (5) Å and (b) between pyrazine (N1B,N3B/C1B,C2B/C6B,C7B, centroid Cg4) and pyridine (N2B/C2B–C6B, centroid Cg5) rings Cg4···Cg5( x, 1/2-y, 1/2+z) 3.6374 (5) Å

The two independent molecules in the asymmetric unit are strikingly similar in respect of their bond distances, bond angles and thermal parameters. For example, for bond distances the average δ/σ = 0.88. The symmetry between molecules A and B is almost that of an inversion centre.

### Experimental

Methyl pyrido[2,3-b]pyrazine-3-carboxylate was synthesized from pyrido[2,3-b] pyrazine-3-carbaldehyde by our recently developed techniques (Goswami & Hazra, 2009; Goswami *et al.*, 2011). Single crystals were grown by slow evaporation of a chloroform solution of the compound, Mp 155–156°C.

### Refinement

All hydrogen atoms were positioned geometrically, with Csp^2^—H = 0.93 Å and C(methyl)—H = 0.96 Å; they were refined using a riding model, with *U*_iso_(H) = x*U*_eq_(C), where x = 1.5 for methyl H and 1.2 for all other H atoms. A rotating group model was applied to the methyl groups.

### Crystal data

**Table:**

C_9_H_7_N_3_O_2_	*F*(000) = 784
*M**_r_* = 189.18	*D*_x_ = 1.519 Mg m^−^^3^
Monoclinic, *P*2_1_/*c*	Mo *K*α radiation, λ = 0.71073 Å
Hall symbol: -P 2ybc	Cell parameters from 6855 reflections
*a* = 9.5135 (1) Å	θ = 2.3–30.1°
*b* = 26.9042 (3) Å	µ = 0.11 mm^−^^1^
*c* = 6.7837 (1) Å	*T* = 100 K
β = 107.686 (1)°	Block, brown
*V* = 1654.24 (4) Å^3^	0.22 × 0.20 × 0.19 mm
*Z* = 8	

### Data collection

**Table:**

Bruker SMART APEXII CCD area-detector diffractometer	4876 independent reflections
Radiation source: fine-focus sealed tube	4118 reflections with *I* > 2σ(*I*)
graphite	*R*_int_ = 0.025
φ and ω scans	θ_max_ = 30.1°, θ_min_ = 2.3°
Absorption correction: multi-scan (*SADABS*; Bruker, 2009)	*h* = −13→12
*T*_min_ = 0.975, *T*_max_ = 0.979	*k* = −36→37
18421 measured reflections	*l* = −8→9

### Refinement

**Table:**

Refinement on *F*^2^	Primary atom site location: structure-invariant direct methods
Least-squares matrix: full	Secondary atom site location: difference Fourier map
*R*[*F*^2^ > 2σ(*F*^2^)] = 0.041	Hydrogen site location: inferred from neighbouring sites
*wR*(*F*^2^) = 0.115	H-atom parameters constrained
*S* = 1.04	*w* = 1/[σ^2^(*F*_o_^2^) + (0.0645*P*)^2^ + 0.3345*P*] where *P* = (*F*_o_^2^ + 2*F*_c_^2^)/3
4876 reflections	(Δ/σ)_max_ < 0.001
255 parameters	Δρ_max_ = 0.38 e Å^−^^3^
0 restraints	Δρ_min_ = −0.30 e Å^−^^3^

### Special details

**Table:**

Experimental. The crystal was placed in the cold stream of an Oxford Cryosystems Cobra open-flow nitrogen cryostat (Cosier & Glazer, 1986) operating at 100.0 (1) K.
Geometry. All s.u.'s (except the s.u. in the dihedral angle between two l.s. planes) are estimated using the full covariance matrix. The cell s.u.'s are taken into account individually in the estimation of s.u.'s in distances, angles and torsion angles; correlations between s.u.'s in cell parameters are only used when they are defined by crystal symmetry. An approximate (isotropic) treatment of cell s.u.'s is used for estimating s.u.'s involving l.s. planes.
Refinement. Refinement of F^2^ against ALL reflections. The weighted R-factor wR and goodness of fit S are based on F^2^, conventional R-factors R are based on F, with F set to zero for negative F^2^. The threshold expression of F^2^ > 2σ(F^2^) is used only for calculating R-factors(gt) etc. and is not relevant to the choice of reflections for refinement. R-factors based on F^2^ are statistically about twice as large as those based on F, and R- factors based on ALL data will be even larger.

### Fractional atomic coordinates and isotropic or equivalent isotropic displacement parameters (Å^2^)

**Table:**

	*x*	*y*	*z*	*U*_iso_*/*U*_eq_	
O1A	0.93918 (7)	0.07303 (3)	0.70643 (12)	0.01812 (16)	
O2A	1.02425 (8)	−0.00559 (3)	0.76407 (12)	0.02050 (17)	
N1A	0.53239 (9)	0.03988 (3)	0.73953 (13)	0.01616 (17)	
N2A	0.58303 (9)	−0.09571 (3)	0.79786 (14)	0.02002 (18)	
N3A	0.74901 (9)	−0.03476 (3)	0.77626 (13)	0.01595 (17)	
C1A	0.66889 (10)	0.05049 (4)	0.74572 (15)	0.01556 (19)	
H1AA	0.6958	0.0836	0.7411	0.019*	
C2A	0.50066 (10)	−0.00925 (3)	0.75265 (14)	0.01429 (18)	
C3A	0.35658 (10)	−0.02404 (4)	0.74564 (15)	0.01738 (19)	
H3AA	0.2817	−0.0007	0.7284	0.021*	
C4A	0.32995 (11)	−0.07339 (4)	0.76471 (16)	0.0193 (2)	
H4AA	0.2361	−0.0843	0.7595	0.023*	
C5A	0.44732 (11)	−0.10778 (4)	0.79259 (16)	0.0209 (2)	
H5AA	0.4274	−0.1411	0.8082	0.025*	
C6A	0.60995 (10)	−0.04658 (4)	0.77600 (15)	0.01502 (18)	
C7A	0.77626 (10)	0.01276 (3)	0.75932 (14)	0.01413 (18)	
C8A	0.92792 (10)	0.02481 (4)	0.74628 (15)	0.01526 (19)	
C9A	1.07833 (11)	0.08837 (4)	0.67914 (17)	0.0203 (2)	
H9AA	1.0750	0.1233	0.6488	0.031*	
H9AB	1.0951	0.0701	0.5668	0.031*	
H9AC	1.1570	0.0819	0.8038	0.031*	
O1B	0.52796 (7)	0.17332 (3)	0.64501 (12)	0.01820 (16)	
O2B	0.45239 (8)	0.25302 (3)	0.63114 (13)	0.02204 (17)	
N1B	0.93475 (9)	0.20808 (3)	0.61052 (13)	0.01529 (17)	
N2B	0.89543 (9)	0.34431 (3)	0.62179 (13)	0.01837 (18)	
N3B	0.72566 (9)	0.28254 (3)	0.61794 (13)	0.01472 (16)	
C1B	0.79969 (10)	0.19717 (3)	0.61024 (15)	0.01489 (18)	
H1BA	0.7715	0.1640	0.6057	0.018*	
C2B	0.96972 (10)	0.25733 (3)	0.61735 (14)	0.01400 (18)	
C3B	1.11389 (11)	0.27225 (4)	0.62514 (15)	0.0176 (2)	
H3BA	1.1861	0.2489	0.6259	0.021*	
C4B	1.14411 (11)	0.32193 (4)	0.63151 (16)	0.0199 (2)	
H4BA	1.2379	0.3330	0.6372	0.024*	
C5B	1.03117 (11)	0.35652 (4)	0.62938 (16)	0.0196 (2)	
H5BA	1.0545	0.3901	0.6336	0.024*	
C6B	0.86440 (10)	0.29472 (3)	0.61752 (14)	0.01401 (18)	
C7B	0.69557 (10)	0.23467 (3)	0.61664 (14)	0.01338 (18)	
C8B	0.54412 (10)	0.22230 (4)	0.62971 (15)	0.01495 (18)	
C9B	0.39006 (11)	0.15787 (4)	0.67601 (18)	0.0215 (2)	
H9BA	0.3901	0.1224	0.6927	0.032*	
H9BB	0.3093	0.1673	0.5581	0.032*	
H9BC	0.3795	0.1736	0.7977	0.032*	

### Atomic displacement parameters (Å^2^)

**Table:**

	*U*^11^	*U*^22^	*U*^33^	*U*^12^	*U*^13^	*U*^23^
O1A	0.0143 (3)	0.0134 (3)	0.0275 (4)	−0.0008 (2)	0.0076 (3)	0.0009 (3)
O2A	0.0153 (3)	0.0168 (3)	0.0304 (4)	0.0025 (3)	0.0085 (3)	0.0018 (3)
N1A	0.0162 (4)	0.0156 (4)	0.0176 (4)	0.0015 (3)	0.0065 (3)	0.0003 (3)
N2A	0.0175 (4)	0.0140 (4)	0.0271 (4)	−0.0009 (3)	0.0047 (3)	0.0023 (3)
N3A	0.0133 (4)	0.0139 (4)	0.0195 (4)	0.0006 (3)	0.0034 (3)	0.0012 (3)
C1A	0.0165 (4)	0.0129 (4)	0.0183 (4)	0.0012 (3)	0.0068 (3)	0.0008 (3)
C2A	0.0142 (4)	0.0154 (4)	0.0132 (4)	0.0008 (3)	0.0040 (3)	0.0001 (3)
C3A	0.0146 (4)	0.0224 (5)	0.0156 (4)	0.0010 (3)	0.0053 (3)	0.0002 (4)
C4A	0.0155 (4)	0.0243 (5)	0.0181 (4)	−0.0044 (4)	0.0050 (3)	0.0000 (4)
C5A	0.0206 (5)	0.0181 (5)	0.0231 (5)	−0.0045 (4)	0.0051 (4)	0.0014 (4)
C6A	0.0142 (4)	0.0137 (4)	0.0164 (4)	0.0006 (3)	0.0035 (3)	0.0012 (3)
C7A	0.0133 (4)	0.0133 (4)	0.0153 (4)	0.0012 (3)	0.0035 (3)	0.0005 (3)
C8A	0.0144 (4)	0.0144 (4)	0.0165 (4)	−0.0004 (3)	0.0040 (3)	−0.0009 (3)
C9A	0.0165 (4)	0.0168 (5)	0.0298 (5)	−0.0020 (3)	0.0101 (4)	0.0011 (4)
O1B	0.0143 (3)	0.0148 (3)	0.0272 (4)	−0.0014 (2)	0.0087 (3)	0.0005 (3)
O2B	0.0166 (3)	0.0183 (4)	0.0338 (4)	0.0029 (3)	0.0116 (3)	0.0028 (3)
N1B	0.0147 (4)	0.0154 (4)	0.0166 (4)	0.0012 (3)	0.0061 (3)	−0.0005 (3)
N2B	0.0211 (4)	0.0140 (4)	0.0215 (4)	−0.0020 (3)	0.0085 (3)	−0.0009 (3)
N3B	0.0146 (4)	0.0145 (4)	0.0159 (4)	0.0010 (3)	0.0060 (3)	0.0004 (3)
C1B	0.0155 (4)	0.0127 (4)	0.0172 (4)	0.0004 (3)	0.0062 (3)	0.0001 (3)
C2B	0.0134 (4)	0.0156 (4)	0.0132 (4)	0.0008 (3)	0.0043 (3)	0.0000 (3)
C3B	0.0138 (4)	0.0217 (5)	0.0177 (4)	−0.0004 (3)	0.0056 (3)	0.0000 (4)
C4B	0.0157 (4)	0.0245 (5)	0.0195 (5)	−0.0054 (4)	0.0052 (4)	−0.0006 (4)
C5B	0.0225 (5)	0.0170 (5)	0.0196 (5)	−0.0053 (4)	0.0068 (4)	−0.0007 (4)
C6B	0.0144 (4)	0.0143 (4)	0.0139 (4)	0.0002 (3)	0.0051 (3)	−0.0002 (3)
C7B	0.0122 (4)	0.0146 (4)	0.0138 (4)	0.0013 (3)	0.0047 (3)	0.0007 (3)
C8B	0.0142 (4)	0.0159 (4)	0.0150 (4)	−0.0004 (3)	0.0048 (3)	0.0007 (3)
C9B	0.0152 (4)	0.0189 (5)	0.0324 (6)	−0.0024 (4)	0.0102 (4)	0.0040 (4)

### Geometric parameters (Å, °)

**Table:**

O1A—C8A	1.3361 (11)	O1B—C8B	1.3344 (11)
O1A—C9A	1.4512 (11)	O1B—C9B	1.4511 (11)
O2A—C8A	1.2066 (12)	O2B—C8B	1.2041 (12)
N1A—C1A	1.3177 (12)	N1B—C1B	1.3174 (12)
N1A—C2A	1.3649 (12)	N1B—C2B	1.3637 (12)
N2A—C5A	1.3212 (13)	N2B—C5B	1.3183 (13)
N2A—C6A	1.3630 (12)	N2B—C6B	1.3648 (12)
N3A—C7A	1.3163 (12)	N3B—C7B	1.3187 (12)
N3A—C6A	1.3601 (12)	N3B—C6B	1.3608 (12)
C1A—C7A	1.4230 (13)	C1B—C7B	1.4239 (13)
C1A—H1AA	0.9300	C1B—H1BA	0.9300
C2A—C3A	1.4141 (13)	C2B—C3B	1.4146 (13)
C2A—C6A	1.4194 (13)	C2B—C6B	1.4200 (13)
C3A—C4A	1.3650 (14)	C3B—C4B	1.3650 (14)
C3A—H3AA	0.9300	C3B—H3BA	0.9300
C4A—C5A	1.4178 (15)	C4B—C5B	1.4183 (15)
C4A—H4AA	0.9300	C4B—H4BA	0.9300
C5A—H5AA	0.9300	C5B—H5BA	0.9300
C7A—C8A	1.5074 (13)	C7B—C8B	1.5074 (13)
C9A—H9AA	0.9600	C9B—H9BA	0.9600
C9A—H9AB	0.9600	C9B—H9BB	0.9600
C9A—H9AC	0.9600	C9B—H9BC	0.9600
			
C8A—O1A—C9A	115.74 (7)	C8B—O1B—C9B	115.14 (8)
C1A—N1A—C2A	116.29 (8)	C1B—N1B—C2B	116.38 (8)
C5A—N2A—C6A	116.72 (9)	C5B—N2B—C6B	116.57 (9)
C7A—N3A—C6A	116.38 (8)	C7B—N3B—C6B	116.36 (8)
N1A—C1A—C7A	121.92 (9)	N1B—C1B—C7B	121.96 (9)
N1A—C1A—H1AA	119.0	N1B—C1B—H1BA	119.0
C7A—C1A—H1AA	119.0	C7B—C1B—H1BA	119.0
N1A—C2A—C3A	120.11 (9)	N1B—C2B—C3B	120.05 (9)
N1A—C2A—C6A	121.57 (8)	N1B—C2B—C6B	121.57 (8)
C3A—C2A—C6A	118.31 (9)	C3B—C2B—C6B	118.38 (9)
C4A—C3A—C2A	118.45 (9)	C4B—C3B—C2B	118.13 (9)
C4A—C3A—H3AA	120.8	C4B—C3B—H3BA	120.9
C2A—C3A—H3AA	120.8	C2B—C3B—H3BA	120.9
C3A—C4A—C5A	119.08 (9)	C3B—C4B—C5B	119.37 (9)
C3A—C4A—H4AA	120.5	C3B—C4B—H4BA	120.3
C5A—C4A—H4AA	120.5	C5B—C4B—H4BA	120.3
N2A—C5A—C4A	124.53 (10)	N2B—C5B—C4B	124.55 (9)
N2A—C5A—H5AA	117.7	N2B—C5B—H5BA	117.7
C4A—C5A—H5AA	117.7	C4B—C5B—H5BA	117.7
N3A—C6A—N2A	116.20 (8)	N3B—C6B—N2B	116.04 (8)
N3A—C6A—C2A	120.92 (9)	N3B—C6B—C2B	120.96 (9)
N2A—C6A—C2A	122.88 (9)	N2B—C6B—C2B	122.98 (9)
N3A—C7A—C1A	122.81 (9)	N3B—C7B—C1B	122.73 (8)
N3A—C7A—C8A	115.56 (8)	N3B—C7B—C8B	115.13 (8)
C1A—C7A—C8A	121.59 (8)	C1B—C7B—C8B	122.11 (8)
O2A—C8A—O1A	124.97 (9)	O2B—C8B—O1B	125.19 (9)
O2A—C8A—C7A	124.05 (9)	O2B—C8B—C7B	123.84 (9)
O1A—C8A—C7A	110.96 (8)	O1B—C8B—C7B	110.94 (8)
O1A—C9A—H9AA	109.5	O1B—C9B—H9BA	109.5
O1A—C9A—H9AB	109.5	O1B—C9B—H9BB	109.5
H9AA—C9A—H9AB	109.5	H9BA—C9B—H9BB	109.5
O1A—C9A—H9AC	109.5	O1B—C9B—H9BC	109.5
H9AA—C9A—H9AC	109.5	H9BA—C9B—H9BC	109.5
H9AB—C9A—H9AC	109.5	H9BB—C9B—H9BC	109.5
			
C2A—N1A—C1A—C7A	1.75 (14)	C2B—N1B—C1B—C7B	0.02 (13)
C1A—N1A—C2A—C3A	−179.51 (9)	C1B—N1B—C2B—C3B	177.88 (9)
C1A—N1A—C2A—C6A	1.27 (13)	C1B—N1B—C2B—C6B	−1.92 (13)
N1A—C2A—C3A—C4A	−178.29 (9)	N1B—C2B—C3B—C4B	179.91 (9)
C6A—C2A—C3A—C4A	0.95 (14)	C6B—C2B—C3B—C4B	−0.28 (14)
C2A—C3A—C4A—C5A	0.57 (14)	C2B—C3B—C4B—C5B	−0.22 (14)
C6A—N2A—C5A—C4A	0.43 (16)	C6B—N2B—C5B—C4B	0.56 (15)
C3A—C4A—C5A—N2A	−1.35 (16)	C3B—C4B—C5B—N2B	0.09 (16)
C7A—N3A—C6A—N2A	−178.69 (9)	C7B—N3B—C6B—N2B	−179.12 (8)
C7A—N3A—C6A—C2A	1.72 (14)	C7B—N3B—C6B—C2B	−0.48 (13)
C5A—N2A—C6A—N3A	−178.34 (9)	C5B—N2B—C6B—N3B	177.50 (8)
C5A—N2A—C6A—C2A	1.24 (15)	C5B—N2B—C6B—C2B	−1.11 (14)
N1A—C2A—C6A—N3A	−3.16 (14)	N1B—C2B—C6B—N3B	2.25 (14)
C3A—C2A—C6A—N3A	177.61 (8)	C3B—C2B—C6B—N3B	−177.55 (9)
N1A—C2A—C6A—N2A	177.28 (9)	N1B—C2B—C6B—N2B	−179.20 (9)
C3A—C2A—C6A—N2A	−1.94 (14)	C3B—C2B—C6B—N2B	0.99 (14)
C6A—N3A—C7A—C1A	1.31 (14)	C6B—N3B—C7B—C1B	−1.43 (13)
C6A—N3A—C7A—C8A	−176.28 (8)	C6B—N3B—C7B—C8B	176.59 (8)
N1A—C1A—C7A—N3A	−3.24 (15)	N1B—C1B—C7B—N3B	1.77 (15)
N1A—C1A—C7A—C8A	174.20 (9)	N1B—C1B—C7B—C8B	−176.12 (9)
C9A—O1A—C8A—O2A	1.37 (14)	C9B—O1B—C8B—O2B	−3.23 (14)
C9A—O1A—C8A—C7A	−176.73 (8)	C9B—O1B—C8B—C7B	175.04 (8)
N3A—C7A—C8A—O2A	−5.04 (14)	N3B—C7B—C8B—O2B	3.54 (14)
C1A—C7A—C8A—O2A	177.34 (9)	C1B—C7B—C8B—O2B	−178.43 (9)
N3A—C7A—C8A—O1A	173.08 (8)	N3B—C7B—C8B—O1B	−174.76 (8)
C1A—C7A—C8A—O1A	−4.54 (12)	C1B—C7B—C8B—O1B	3.27 (12)

### Hydrogen-bond geometry (Å, °)

**Table:**

*D*—H···*A*	*D*—H	H···*A*	*D*···*A*	*D*—H···*A*
C3A—H3AA···O2A^i^	0.93	2.54	3.2401 (13)	133.
C4A—H4AA···N2B^ii^	0.93	2.55	3.3311 (14)	141.
C9A—H9AA···N1B	0.96	2.62	3.4741 (14)	149.
C3B—H3BA···O2B^iii^	0.93	2.53	3.2496 (14)	135.
C4B—H4BA···N2A^iv^	0.93	2.51	3.3350 (14)	147.
C9B—H9BA···N1A	0.96	2.57	3.4266 (14)	149.

Symmetry codes: (i) *x*−1, *y*, *z*; (ii) −*x*+1, *y*−1/2, −*z*+3/2; (iii) *x*+1, *y*, *z*; (iv) −*x*+2, *y*+1/2, −*z*+3/2.
